# Longitudinal analysis of behavioral addictions among children and adolescents in the context of the COVID-19 pandemic

**DOI:** 10.3389/fpsyg.2026.1791527

**Published:** 2026-05-26

**Authors:** Shaked Farbstein-Yavin, Anat Shoshani, Yari Gvion, Ariel Kor

**Affiliations:** 1 Department of Psychology, Bar-Ilan University, Ramat-Gan, Israel; 2 Baruch Ivcher School of Psychology, Reichman University, Herzliya, Israel

**Keywords:** behavioral addictions, children and adolescents, COVID-19 pandemic, developmental pathways, longitudinal study, risk and protective factors, youth mental health

## Abstract

**Introduction:**

Behavioral addictions in childhood and adolescence remain understudied, particularly beyond digital media use. The COVID-19 pandemic introduced substantial disruptions to youths' daily routines, social functioning, and emotional well-being, potentially increasing vulnerability to maladaptive behavioral patterns. This three-wave longitudinal study examined trajectories and correlates of gambling, shopping, exercise, and eating addiction symptoms among children and adolescents across the pandemic period.

**Methods:**

The study included 1,665 Israeli students aged 9-16.7 years assessed before the COVID-19 pandemic (October 2019), after the first wave (November 2020), and after the fifth wave and return to regular schooling (April 2022). Participants completed validated measures of behavioral addiction symptoms, psychiatric symptoms, negative and positive affect, sensation seeking, life satisfaction, social support, secure attachment, hope, future orientation, grit, and gratitude. Structural equation modeling tested indirect effects of protective factors on behavioral addictions via risk factors at baseline, and linear growth-curve models estimated change in addiction symptoms over time.

**Results:**

Symptoms of exercise, gambling, and shopping addictions increased significantly across the three time points, whereas eating addiction symptoms remained stable. Negative affect, psychiatric symptoms, and sensation seeking predicted higher levels of behavioral addiction, and social support was unexpectedly associated with higher exercise, gambling, and eating addiction symptoms. In contrast, future orientation, life satisfaction, hope, positive emotions, and secure attachment were linked to fewer symptoms. Protective factors were related to lower behavioral addiction symptoms only indirectly, through their associations with reduced negative affect and psychiatric symptoms, indicating full mediation. Girls reported more shopping and eating addiction symptoms, whereas boys reported more gambling and exercise symptoms; older age was linked to fewer shopping, exercise, and eating symptoms but more gambling symptoms.

**Discussion:**

These findings suggest that youth behavioral addictions intensified during and after the pandemic, even when most symptoms remained subclinical. Emotional and social resources functioned as resilience factors primarily by shaping mental health, highlighting negative affect and psychiatric distress as key intervention targets to prevent behavioral addictions in children and adolescents.

## Introduction

Addictive behaviors among children and adolescents have become a growing global public concern, as they are increasingly recognized as patterns of repetitive behavior driven by an overwhelming urge to engage in certain activities despite adverse consequences and with significant distress or impairment in personal, social, educational, or occupational functioning (e.g., Bećirović and Pajević, 2020; Simón Márquez et al., 2025). Similar to substance use disorders, reward-seeking behaviors such as gambling, shopping, and screen use can, when carried out excessively, involve repeated difficulties in resisting urges and continued engagement despite adverse outcomes, leading to impaired daily functioning, negative social consequences, and considerable psychological distress (e.g., Brand et al., 2025; Vidal and Meshi, 2023).

Despite increasing frequency and public concern, most empirical work on youth addictive behaviors still predominantly focuses on substances and digital media use, whereas other proposed addictive behaviors in childhood and adolescence–such as exercise addiction, shopping addiction, and eating addiction–remain less clearly established and less extensively characterized, particularly in younger populations (e.g., Laurent and Sibold, 2016; Simón Márquez et al., 2025; Somma et al., 2023; Vidal and Meshi, 2023). It is therefore important to examine these specific addictions in today's children and adolescents, particularly in the context of the COVID-19 pandemic, to better understand behavioral addictions in youth and their associations with selected psychological risk and protective factors during this period. Gambling disorder was the first behavioral addiction formally recognized in DSM-5, with diagnostic criteria paralleling those of substance use disorders (American Psychiatric Association, 2013). However, the extent to which other behaviors constitute standalone addictive disorders remains under debate and requires further investigation, with ongoing efforts to refine diagnostic criteria for such conditions (Potenza et al., 2018; Brand et al., 2020).

The COVID-19 pandemic provides an important context in which to study the development of behavioral addictions in youth. Although children and adolescents were less affected medically by the disease itself, they were exposed to multiple stressors that markedly influenced their well-being, including heightened fear of infection, boredom, frustration, and reduced privacy at home, financial strain, and family conflict (Brooks et al., 2020; Meade, 2021; Shoshani and Kor, 2022). Prolonged lockdowns, school closures, and restrictions on extracurricular activities substantially changed daily routines, leading many young people to spend more time indoors and online, reduce their physical activity, and experience disruptions in eating habits. These changes were also associated with increases in various problematic behaviors.

Evidence on the pandemic's effects on youth health-related behaviors supports these concerns but has rarely focused directly on behavioral addictions. Studies from the United States and Europe have documented increases in body mass index and obesity, as well as higher consumption of calorie-dense snacks among children and adolescents during COVID-19 (Woolford et al., 2021; Jenssen et al., 2021; Farello et al., 2022). A scoping review of children aged 5–17 years reported reduced physical activity and increased sedentary behavior during the pandemic (Paterson et al., 2021). At the same time, several studies have described substantial increases in screen use among youth (e.g., Pardhan et al., 2022). However, there is still a notable lack of research tracking changes in gambling and shopping behaviors among children and adolescents during and after the pandemic, despite the increased accessibility of online gambling and shopping platforms in this period. Given these trends, it is reasonable to hypothesize that gambling and shopping behaviors in youth also intensified during COVID-19.

Beyond documenting behavioral changes, examining selected risk and protective factors associated with gambling, shopping, exercise, and eating addictions in children and adolescents is crucial for designing targeted prevention and intervention strategies. Prior work suggests that a range of emotional and psychological characteristics–such as psychiatric symptoms, negative affect, and sensation seeking–may heighten vulnerability to addictive behaviors. In contrast, life satisfaction, positive affect, gratitude, future orientation, grit, hope, secure attachment, and social support may function as protective resources. However, relatively few studies have investigated how these selected factors jointly predict excessive gambling, shopping, exercise, and eating behaviors or the presence of addiction symptoms in youth. Moreover, many existing studies conducted during COVID-19 lack pre-pandemic measurements or long-term follow-up, limiting the ability to determine how the pandemic altered developmental trajectories of behavioral addictions.

To address these gaps, the present study followed children and adolescents over a three-year period, beginning several months before the onset of the COVID-19 pandemic and extending to 1 year after the resumption of regular schooling. This longitudinal design makes it possible to examine how symptoms of gambling, shopping, exercise, and eating addictions changed from pre-pandemic baseline through different phases of the pandemic, and to identify risk and protective factors associated with these trajectories. By doing so, the study aims to clarify not only the acute impact of COVID-19 on youth behavioral addictions but also the longer-term effects of pandemic-related disruptions on addictive behaviors and mental health in this population.

### The present study

The present study investigated associations between selected, theoretically informed risk and protective factors and symptoms of gambling, shopping, exercise, and eating addictions among children and adolescents using a three-wave longitudinal design spanning approximately 3 years, including assessments before and during the COVID-19 pandemic. The study focused on key risk factors–psychiatric symptoms, negative affect, and sensation seeking–and a broad set of protective factors, including life satisfaction, positive affect, gratitude, future orientation, grit, hope, secure attachment, and perceived social support. Repeated measurements at multiple time points allowed us to examine within-person changes in behavioral addictions and mental health across distinct stages of the pandemic.

Hypothesis 1 proposed significant increases in eating, exercise, shopping, and gambling addiction symptoms across the study period relative to the pre-pandemic baseline. Hypothesis 2 anticipated that higher levels of psychiatric symptoms, negative affect, and sensation seeking would be associated with greater increases in addiction symptoms. In contrast, higher levels of positive affect, life satisfaction, social support, secure attachment, hope, future orientation, grit, and gratitude would be linked to smaller increases. Exploratory analyses examined whether age, gender, and other demographic variables predicted differences in levels and changes of addictive behavior symptoms over time. Hypothesis 3 further proposed that risk factors would mediate the associations between protective factors and addiction symptoms, such that higher protective resources would be related to lower psychiatric symptoms and negative affect, which in turn would predict fewer gambling, shopping, exercise, and eating addiction symptoms.

## Materials and methods

### Participants

The research sample consisted of 1,665 students in grades four to 10, aged 9 to 16.7 years, with an average age of 12.10 years (*SD* = 2.25). Among them, 830 were male, and 835 were female. Participants were selected from a pool of 18 schools in three distinct geographical regions of Israel. The six schools included in the study were chosen randomly and were representative of geographic location, socioeconomic status (SES), and school type. Special education and ultra-orthodox religious institutions were excluded from the sample. The recruitment of schools was facilitated through collaboration with municipal education departments.

Thirteen students chose not to participate, and nine were excluded due to incomplete data. Participants' questionnaires were coded for confidentiality. Data collection took place at three-time points: pre-COVID-19 outbreak (October 2019; *N* = 1665), after the initial wave of the pandemic (November 2020; 12-month follow-up; *N* = 1,628), and after the fifth wave (April 2022; 30-month follow-up; *N* = 1,624).

Regarding participants' religious affiliation, 95% were identified as Jewish, 4% as Christian, and 1% as Muslim. Regarding socioeconomic status, 18.4% belonged to high-status families, 64.1% to medium-status families, and 17.5% to low-status families. Concerning family structure, 72.1% of participants had married parents, while 22.8% had divorced or separated parents. Furthermore, 5.1% of participants came from single-parent households, were unmarried parents, or were raised by widowed parents. The completion of questionnaires took place on tablets provided during regular school periods. Table 1 presents the demographic and baseline characteristics of the study sample at baseline.

**Table 1:** Sample characteristics at baseline.

Characteristic	*n* (%)
Age, Mean (SD)	12.10 (2.25)
School Grades	
4th Grade	225 (13.5%)
5th Grade	235 (14.1%)
6th Grade	230 (13.8%)
7th Grade	238 (14.3%)
8th Grade	245 (14.7%)
9th Grade	250 (15.0%)
10th Grade	242 (14.6%)
Gender	
Girls	835 (50.2%)
Boys	830 (49.8%)
Religion	
Jewish	1581 (95%)
Muslim	17 (1%)
Christian	67 (4%)
Socioeconomic status	
Low SES	291 (17.5%)
Middle SES	1067 (64.1%)
High SES	307 (18.4%)
Family status	
Married	1200 (72.1%)
Divorced/Separated	380 (22.8%)
Single	85 (5.1%)

*N* = 1,665; T1 - Baseline, October 2019.

### Measures

#### Multi-Dimensional Addiction Questionnaire

Exercise, Shopping, Gambling, and Eating, we used a multi-dimensional questionnaire developed for the current study to capture both behavioral frequency and psychological features associated with addictive behavior. The questionnaire was based on items from the Gaming Addiction Identification Test (GAIT; Vadlin et al., 2015), which used the criteria for Gambling Disorder suggested by the DSM-5 (American Psychiatric Association, 2013) and (Griffiths 2005) six core components of addiction (e.g., salience, withdrawal, and relapse) and has demonstrated strong internal consistency and content validity in prior research. Regarding each type of addiction, participants were shown four items: (1) The first item measured frequency and duration of use, and participants are asked to rate it on a five-point Likert scale (e.g., Never, 1-2 a week, 3-4 a week, Every day, More than once a day); (2) The remaining three items assessed addiction-related characteristics–namely withdrawal, relapse, and reduced interest in other activities, and participants are asked to rate each on a five-point Likert scale (1 = Never, to 5 = Usually). Thus, the questionnaire assessed not only the intensity of engagement, but also psychological indicators commonly associated with addictive patterns. The original GAIT questionnaire demonstrated good content validity, with mean scores ranging from 0.97 to 0.99. In the current sample, Cronbach's alpha coefficients ranged from 0.67 to 0.78.

#### The brief symptom inventory 18 (BSI-18)

The BSI-18 (Derogatis, 2000) comprises 18 items and assesses mental and psychological distress and comorbidities in patients with different mental and somatic illnesses. The 18 items compose three dimensions: (1) Somatization; (2) Depression; and (3) Anxiety; and a comprehensive measure of overall psychological distress called the Global Severity Index (GSI), which is derived by summing the distress ratings assigned to each item. Each subscale of the 18 items contains six items from the three equivalent dimensions of the BSI-18. Participants were asked to rate each on a five-point Likert scale (0 = not at all, to 4 = very much). The Cronbach's alphas for this scale ranged from 0.82 to 0.88.

#### The positive and negative affect schedule for children (PANAS-C)

The PANAS-C (Ebesutani et al., 2012) is a questionnaire consisting of 10 items designed to assess the subjective well-being of children and adolescents by measuring positive and negative emotions experienced in the previous month. It includes five items related to negative emotions (e.g., upset, fearful, and anxious) and five related to positive emotions (e.g., excited, proud, and enthusiastic). Participants rated their experiences on a 5-point Likert-type scale, ranging from 1 (very slightly or not at all) to 5 (extremely). Negative Affect (NA) and Positive Affect (PA) scores are calculated by summing the relevant item scores, with alpha coefficients of 0.80–0.87 for PA and 0.79–0.84 for NA.

#### Impulsivity and sensation seeking

To examine students' impulsivity and sensation-seeking in the present study, we integrated two questionnaires, the Impulsivity and Sensation Seeking Scale (Harden and Tucker-Drob, 2011) and Arnett's Inventory of Sensation Seeking (AISS; Arnett, 1994). (Harden and Tucker-Drob 2011) self-report questionnaire draws on multiple questionnaires (NLSY79 Children and Young Adults, 2009). It contains six items assessing impulsivity and sensation-seeking. Participants were asked to rate each on a four-point Likert scale (1 = Strongly Disagree, to 4 = Strongly Agree); from Arnett's Inventory of Sensation Seeking (AISS; Arnett, 1994), we use two items. Participants were asked to rate each item on a four-point Likert scale (1 = Do Not Agree At All, to 4 = Totally Agree). The scale demonstrated high internal consistency with Cronbach's alphas ranging from 0.88 to 0.93.

#### The Brief Multidimensional Students' Life Satisfaction Scale (BMSLSS)

The BMSLSS (Seligson et al., 2003) is a self-report questionnaire that measures life satisfaction in various domains. Each item assesses satisfaction in life in one of five domains (i.e., “I would describe my satisfaction with my family life/ friendships/ school experience/ myself/ where I live as...”). It contains five items, and participants were asked to rate each on a seven-point Likert scale (1 = Terrible, to 7 = Delighted). The Cronbach's alphas ranged from 0.83 to 0.87.

#### The Multidimensional Scale of Perceived Social Support-Adapted Questionnaire (MSPSS-Adapted)

The MSPSS-Adapted (Frison and Eggermont, 2015), an adaptation of the Multidimensional Scale of the Perceived Social Support Questionnaire (Zimet et al., 1988), is a self-report questionnaire developed to measure the perceived levels of social support acquired from friends. The questionnaire consists of 4 items (e.g., “My friends really try to help me”), all of which begin with the clause: “When you are feeling down or in a difficult situation…”; participants were asked to rate each on a five-point Likert scale (1 = strongly disagree, to 5 = strongly agree). The Cronbach's alphas ranged from 0.84 to 0.90.

#### Attachment Style Classification Questionnaire (ASCQ)

We utilized the Hebrew version by (Mikulincer et al. 1990) from the questionnaire developed by (Hazan and Shaver 1987) to classify attachment styles in adults. The ASCQ (Finzi et al., 2000) comprises 15 items, which form three factors that correspond to Ainsworth's three attachment patterns: (1) secure; (2) anxious/ambivalent; and (3) avoidant. Participants were asked to rate each item on a five-point Likert scale (1 = Not at all, to 5 = Very much). The scale demonstrated high Cronbach's alphas ranging from 0.85 to 0.91.

#### The Children Hope Scale

We used (Lackaye et al. 2006) Hebrew adaptation of The Children's Hope Scale (Snyder et al., 1997). It is a self-report questionnaire aimed to measure the belief in one's capabilities to achieve proper routes to goals, along with the self-related beliefs toward establishing and maintaining movement toward them. This measure consists of 6 items, assigned into two subscales: (1) agency; and (2) pathways. Participants were asked to rate each item on a six-point Likert scale (1 = None of the time, to 6 = All of the time). The Cronbach's alphas for this scale ranged from 0.86 to 0.91.

#### Design My Future (DMF)

The DMF (Di Maggio et al., 2016) is a self-report questionnaire designed to assess youths' future orientation and resilience. The full-length questionnaire comprises 19 items, which form two factors: (1) Future Orientation and (2) Resilience. The present study utilizes an abbreviated version of the questionnaire, comprising eight items, to focus on the Future Orientation construct. Participants were asked to rate each item on a five-point Likert scale (1 = It describes me not at all, to 5 = It describes me very well). The Cronbach's alphas for this scale ranged from 0.90 to 0.92.

#### The Short Grit Scale (Grit–S)

The Grit-S (Duckworth and Quinn, 2009) is a shortened version of the Grit Scale self-report questionnaire (Duckworth et al., 2007). It is a self-report questionnaire designed to measure trait-level perseverance and passion for long-term goals. It comprises eight items, which form two factors: (1) consistency of interest; and (2) perseverance of effort. Participants were asked to rate each item on a five-point Likert scale (1 = not like me at all, to 5 = very much like me). The Cronbach's alphas for this scale ranged from 0.91 to 0.94.

#### Gratitude Questionnaire-6 (GQ-6)

The GQ-6 (McCullough et al., 2002) is a self-report questionnaire used for measuring a grateful disposition. It is comprised of six items (e.g., “If I had to list everything that I felt grateful for, it would be a very long list”), and participants were asked to rate each on a seven-point Likert scale (1 = Strongly disagree, to 7= Strongly agree). Past research has found item 6, “Long amounts of time can go by before I feel grateful to something or someone,” to be “difficult to understand,” “very abstract,” for students, and consequently inappropriate for youth (Froh et al., 2011). The present study will, therefore, refrain from making use of it. The Cronbach's alphas for this scale ranged from 0.82 to 0.85.

### Procedure

Following the approval of the Israeli Ministry of Education and the relevant academic ethics committee (Approval No. 12044), detailed information regarding the research objectives was provided to the participant's parents. Both parents and students were requested to provide written informed consent for the student's involvement in the study. Data collection took place within the school premises at three-time points: Before the COVID-19 pandemic outbreak (October 2019), after the first wave of the pandemic (November 2020), and after the fifth pandemic wave (April 2022). The questionnaires were completed electronically by the participants in their classrooms during regular school hours, using Android tablets equipped with the Qualtrics Offline application. Participants were informed that their involvement in the study was voluntary and that their responses would be kept confidential.

### Statistical analysis

A structural equation model (SEM) was conducted using Amos 28 to examine the indirect effects of protective factors on behavioral addiction symptoms, via risk factors, at baseline. Model specification followed a two-step procedure. First, we estimated a measurement model that included all hypothesized indicators for the three latent factors: a protective-factors factor (future orientation, grit, hope, social support, life satisfaction, positive affect, and gratitude), a risk-factors factor (psychiatric symptoms, negative affect, and sensation seeking), and a behavioral-addiction factor (shopping, gambling, eating, and exercise addiction symptoms). Indicators with standardized factor loadings below 0.40 on their intended latent factor were considered for removal, consistent with conventional recommendations for item retention in confirmatory factor analysis (Brown, 2015). Removal decisions were made sequentially, one indicator at a time, and were retained only when the resulting factor remained theoretically coherent and when overall model fit improved. Based on these criteria, gratitude was removed from the protective-factors factor, sensation seeking was removed from the risk-factors factor, and exercise addiction was removed from the behavioral-addiction factor (all loadings < 0.40). The final measurement model was then used as the basis for estimating the structural mediation model. Indirect effects were estimated using bias-corrected bootstrapping with 5,000 resamples. Model fit was evaluated using multiple indices, following (Hu and Bentler 1999) recommendations: RMSEA (values ≤ 0.08 indicating acceptable fit), SRMR ( ≤ 0.08 acceptable), and CFI and TLI (≥0.90 acceptable, ≥0.95 good).

Mixed-model growth curve analyses were then used to investigate changes in addictive behavior outcomes from baseline to the third measurement point. A trend analysis was conducted to examine patterns of change over time, indicating that linear trend models provided the best fit for the outcome variables. Curve models were fitted for the different outcomes, with intercept and slope specified as model parameters. An unstructured variance-covariance matrix was used.

The Level 1 models examined growth trajectories as a function of time and the risk and protective factors, capturing within-person change across the assessment points. The Level 2 models examined whether between-person demographic variables accounted for variation in the Level 1 growth parameters. Gender and secure attachment were entered using dummy coding, and age was centered around the sample mean. As part of the analytic procedure, quadratic age effects and age interactions with key predictors were also examined to determine whether a more complex age specification was warranted. These effects were not statistically significant and did not materially change the pattern of findings; therefore, age was retained as a linear covariate in the final models. Additional demographic variables were tested but were not significant predictors. Several covariates, including class and school effects, school size, and socioeconomic composition, were also considered to examine the influence of school- and classroom-level characteristics on the outcome variables. However, these effects were not significant and were therefore not included in the final models. *T*-tests and chi-square tests were used to examine mean differences over time, and Cohen's *d* was calculated as an index of effect size. Missing data, which comprised less than 3% of the study variables, were handled using maximum likelihood estimation.

## Results

### Descriptive statistics

Table 2 displays the mean and standard deviation for the study variables at the three measurement points. The normality assumption was confirmed based on the skewness and kurtosis tests. At baseline, 1.8%, 3.4%, 3.2% and 0.3% reported frequent shopping, eating, exercise and gambling addiction symptoms, respectively.

**Table 2:** Means and standard deviations of outcome measures at the three measurement points.

Variable	T1 *n* = 1665		T2 *n* = 1628		T3 *n* = 1624		T1 vs T3	*d*
	Mean	*SD*	Mean	*SD*	Mean	*SD*	*t/*χ^2^	
Sport addiction symptoms	1.74	0.55	1.84	0.58	4.65	5.62	*t* = −20.98^***^	0.73
Shopping addiction symptoms	1.58	0.77	1.64	0.82	1.69	0.83	*t* = −3.88^***^	0.14
Gambling addiction symptoms	1.18	0.42	1.23	0.62	1.82	0.94	*t* = −15.09^***^	0.85
Eating addiction symptoms	1.81	0.83	1.85	0.82	1.85	0.87	*t* = −1.25	0.04
Gratitude	2.57	0.78	2.32	0.92	2.64	0.94	*t* = −2.24^*^	0.08
Future orientation	3.83	0.87	3.83	0.90	3.78	1.00	*t* = 1.55	0.05
Grit	2.98	0.69	2.94	0.73	3.06	0.70	*t* = −3.15^***^	0.11
Hope	25.45	7.03	25.47	7.25	23.24	9.32	*t* = 7.60^***^	0.27
Social support	15.13	4.29	14.90	4.28	14.94	4.61	*t* = 1.23	0.04
LS	18.80	4.92	19.13	4.97	21.25	5.07	*t* = −13.48^***^	0.49
PA	18.15	4.24	18.89	4.43	15.72	8.15	*t* = 10.74^***^	0.37
NA	9.46	3.85	9.74	4.12	11.68	7.26	*t* = −10.98^***^	0.38
Sensation seeking	19.96	5.36	19.68	5.63	18.03	5.57	*t* = 9.80^***^	0.35
Psychiatric symptoms (GSI)	8.30	10.35	8.63	8.33	13.00	10.01	*t* = −13.19^***^	0.46

T1- October 2019; T2 - November 2020; T3 – April 2022; LS, Life satisfaction; PA, Positive affect; NA, Negative affect. *p* < 0.05^*^, *p* < 0.01^**^, *p* < 0.001^***^.

Girls reported more involvement in shopping, *t* (1664) = 5.18, *p* < 0.001, and emotional eating episodes, *t* (1664) = 3.19, *p* < 0.001, than boys, while boys reported more sports participation, *t* (1664) = 4.42, *p* < 0.001, and playing gambling games than girls, *t* (1664) = 3.19, *p* < 0.001.

### Risk and protective factors for behavioral addiction symptoms

**Figure 1:**
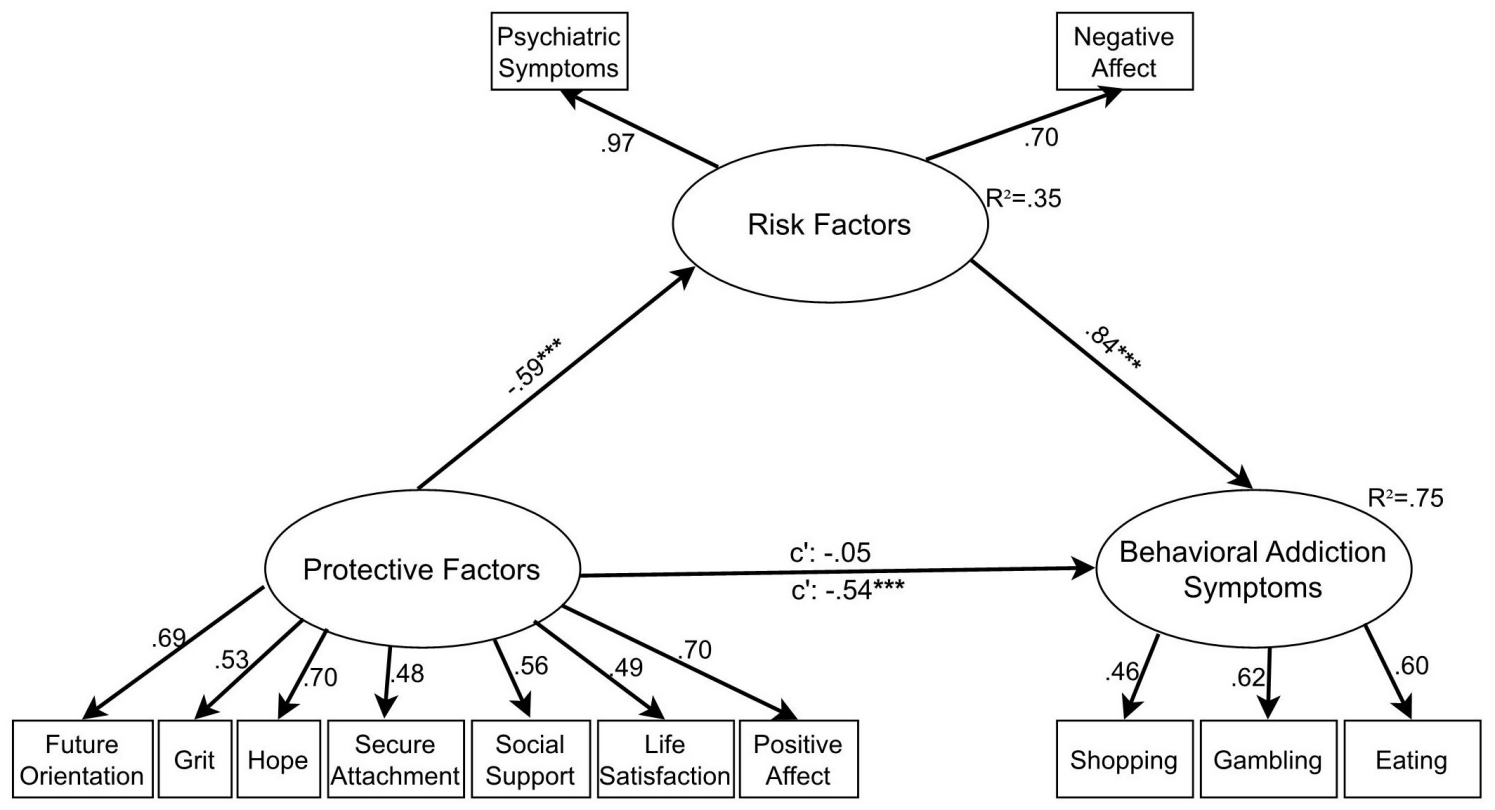
The direct and indirect effects of protective factors through risk factors on behavioral addiction symptoms; ^***^p < 0. 001.

The final structural model included protective factors as the predictor, risk factors as the mediator, and behavioral addiction symptoms as the outcome (Figure 1). The model fit indices indicated acceptable overall fit: χ^2^(48) = 744.23, *p* < 0.001; RMSEA = 0.093; SRMR = 0.078; CFI = 0.90; TLI = 0.88. As is common with large samples (N = 1,665), the χ^2^ test was significant and should be interpreted with caution, because even trivial discrepancies between model and data yield significant χ^2^ values at this sample size (Kline, 2016). SRMR and CFI met conventional thresholds, whereas RMSEA slightly exceeded the 0.08 cutoff and TLI fell just below 0.90, indicating that although the model provides a reasonable representation of the data, some residual unexplained covariance remains.

As hypothesized, the protective factors were negatively associated with behavioral addiction symptoms (β = −0.59, SE = 0.05, *p* < 0.001), while the risk factors were positively associated with behavioral addiction symptoms (β = 0.84, SE = 0.04, *p* < 0.001).

Furthermore, the indirect effect of the protective factors on behavioral addiction symptoms, through the risk factors was significant (B = −0.50; SE = 0.04; 95% CI = −0.573−0.425, *p* < 0.001). This result indicates that the protective factors were associated with lower risk factors, which in turn were associated with lower behavioral addiction symptoms. The direct relationship between the protective factors and behavioral addiction symptoms was not significant (c': β = −0.05, SE = 0.05, *p* = 0.32), suggesting full mediation by the mediator.

### Changes in addictive behavior symptoms and mental health

**Figure 2:**
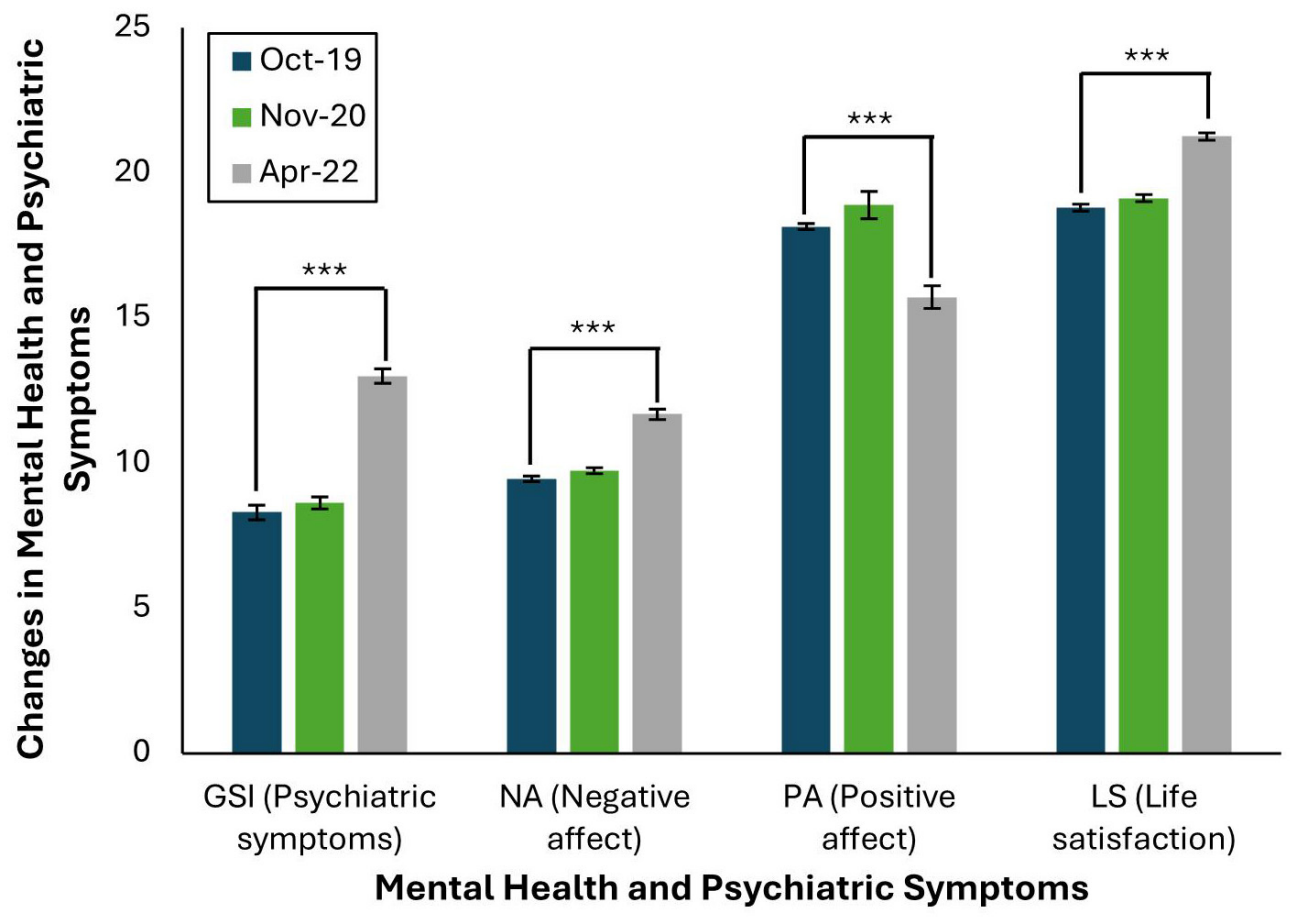
Changes in mental health and psychiatric symptoms throughout the COVID-19 pandemic. ***p < 0.001.

Between the first and third measurement points of the study, there was a significant increase in symptoms of addiction to exercise (*t* = −20.98, *p* < 0.001), gambling (*t* = −15.09, *p* < 0.001) and shopping (*t* = −3.88, *p* < 0.001), but not in eating addiction symptoms (*t* = −1.25, *p* = 0.18). Regarding mental health measures, there was a significant increase in negative emotions and psychiatric symptoms. In contrast, there was an improvement in gratitude, life satisfaction, and grit, while hope and positive emotions decreased. Figure 2 illustrates the changes in mental health indicators across the study period.

The estimated fixed effects for the growth linear models are presented in Table 3. The unconditional mean models have demonstrated low ranges of ICC (0.01-0.25) for addictive behaviors during the study course. This indicates that the within-person stability in these variables was relatively low, with most of the total variability stemming from annual changes in addictive behaviors.

**Table 3:** Trends in behavioral addiction symptoms from time 1 to time 3.

Parameter		Sport addiction symptoms		Shopping addiction symptoms		Gambling addiction symptoms		Eating addiction symptoms	
	Fixed effects	Coefficient (95% CI)	*SE*	Coefficient (95% CI)	*SE*	Coefficient (95% CI)	*SE*	Coefficient (95% CI)	*SE*
	Intercept	0.91^***^ (0.45-1.37)	0.24	1.30^***^ (1.11-1.48)	0.09	0.99^***^ (0.79-1.20)	0.10	1.77^***^ (1.58-1.96)	0.10
Level 1	Time	0.62^***^ (0.52-0.72)	0.05	0.03^*^ (−0.06-0.003)	0.02	0.23^***^ (0.19-0.28)	0.02	0.05^**^ (0.02-0.08)	0.02
	Gratitude	0.10^**^ (0.04-0.16)	0.03	−0.01 (−0.04-0.01)	0.01	0.01 (−0.02-0.05)	0.02	−0.001 (−0.03-0.03)	0.01
	Future orientation	0.19^***^ (0.11-0.27)	0.04	−0.06^***^ (−0.10–0.03)	0.02	−0.06^**^ (−0.10–0.02)	0.02	−0.04^*^ (−0.07–0.008)	0.02
	Grit	0.45^***^ (0.36-0.54)	0.05	0.04^**^ (0.006-0.08)	0.02	−0.02 (−0.07-0.02)	0.02	0.01 (−0.03-0.05)	0.02
	Hope	−0.05^***^ (-0.06–0.04)	0.01	0.00 (−0.002-0.006)	0.002	0.002 (−0.003-0.007)	0.003	−0.004 (−0.008-0.001)	0.002
	Social support	0.07^***^ (0.05-0.08)	0.01	0.02 (0.01-0.02)	0.003	0.01^***^ (0.005-0.02)	0.003	0.02^***^ (0.02-0.03)	0.003
	LS	0.12^***^ (0.10-0.13)	0.01	−0.01^***^ (−0.01–0.004)	0.003	0.01 (−0.005-0.006)	0.003	−0.02^***^ (−0.03–0.02)	0.003
	PA	−0.23^***^ (−0.25–0.22)	0.01	−0.002 (−0.007-0.004)	0.003	−0.02^***^ (−0.03–0.02)	0.004	−0.01 (−0.01-0.001)	0.003
	NA	0.12^***^ (0.11-0.14)	0.01	0.02^***^ (0.02-0.03)	0.003	0.02^***^ (0.02-0.03)	0.003	0.01^***^ (0.009-0.02)	0.003
	Sensation seeking	−0.06^***^ (−0.07–0.05)	0.01	0.004^*^ (0.000-0.008)	0.002	0.001 (−0.009-0.000)	0.002	0.01^***^ (0.009-0.02)	0.002
	Psychiatric symptoms (GSI)	0.04^***^ (0.03-0.05)	0.00	0.02^***^(0.02-0.02)	0.002	0.01^***^ (0.006-0.01)	0.002	0.02^***^ (0.02-0.03)	0.002
Level 2	Age	−0.12^***^ (-0.14–0.09)	0.01	−0.03^***^ (−0.04–0.02)	0.01	0.08^***^ (0.07-0.10)	0.01	−0.03^***^ (−0.04–0.02)	0.01
	Gender (Boys)	0.34^***^ (0.21-0.46)	0.06	−0.09^***^ (−0.13–0.04)	0.02	0.06 (−0.003-0.12)	0.03	−0.02 (−0.06-0.03)	0.02
	Secure attachment	0.06 (0.08-0.20)	0.07	−0.09^*^ (−0.15–0.04)	0.03	0.01 (−0.05-0.08)	0.03	−0.08^**^ (−0.13–0.02)	0.03
Variance components	Within person	0.18^***^		0.63^***^		0.10^***^		0.49^***^	
	Between person	0.08^***^		0.09^***^		0.02		0.01	
Proportion explained	R^2^ within	0.62		0.29		0.58		0.28	
	R^2^ between	0.07		0.13		0.01		0.50	
	ICC	0.25		0.24		0.03		0.04	

*N* = 1,665; LS, Life satisfaction; PA, Positive affect; NA, Negative affect. *p* < 0.05^*^, *p* < 0.01^**^, *p* < 0.001^***^.

The study participants exhibited notable growth in symptoms associated with exercise, shopping, and gambling addictions. However, the occurrence of these symptoms did not escalate to the level of an addiction. By the time of the third evaluation, only a minority of participants experienced these symptoms frequently, with 2.4% in shopping, 3% in gambling, and 2.9% in eating behaviors.

The Level 1 models investigated various potential risk and protective factors related to addiction symptoms. Several protective factors for addiction behaviors were identified. Future orientation was associated with fewer symptoms of shopping, gambling, and eating addiction. Life satisfaction was linked to lower addiction symptoms to shopping and eating. Hope was associated with decreased exercise addiction symptoms. Positive emotions were associated with less addiction to exercise and gambling.

Furthermore, several risk factors for addiction behaviors were identified. Sensation-seeking and psychiatric symptoms were linked to more shopping and eating addiction symptoms. Negative emotions were associated with more exercise, shopping, gambling, and eating addiction symptoms. Contrary to our expectation, social support was a risk and not a protective factor associated with more exercise, gambling, and eating addiction symptoms.

After incorporating the level 2 variables of gender, age, and attachment patterns into the models, significant effects were observed. Symptoms of addiction to shopping, exercise, and eating decreased with age, except for gambling addiction. Boys reported lower levels of shopping addiction but higher levels of exercise addiction than girls. Secure attachment was identified as a protective factor associated with reduced shopping and eating addiction symptoms.

## Discussion

The present study examined addictive behavior symptoms related to gambling, shopping, physical exercise, and eating in children and adolescents and the impact of the COVID-19 pandemic on these behaviors. We also examined how selected risk and protective factors were associated with these behaviors from the beginning of the 2019 academic year, prior to the outbreak of the COVID-19 pandemic, until April 2022, 1 year after students returned to regular school routines.

The findings suggest a significant increase in addiction symptoms related to gambling, shopping, and physical exercise over the study period. Studies examining changes in behavioral addictions (namely, gambling, shopping, exercise, and eating) during the COVID-19 pandemic among children and adolescents are limited, and the impact of the pandemic on addictive behaviors in this population is not yet fully understood. Regarding gambling, studies on older populations mainly reported decreased or no change in addiction behavior due to lockdown restrictions (e.g., Brodeur et al., 2021). However, a study conducted on young adults in the UK found an increase in online gambling frequency during the pandemic (Emond et al., 2022). An emerging argument highlights the blurring lines between gambling and gaming activities (Kim and King, 2020). This view may suggest that the social aspects of gaming, which surged during COVID-19, have fostered a sense of community and belonging, particularly during the pandemic when in-person social interactions were limited. This online environment may also serve as a gateway from gaming to online gambling, as young individuals could be exposed to gambling-related content or advertisements while participating in gaming activities.

Regarding shopping addiction, a study conducted in Canada found that many participants reported increased physical and online shopping, including non-essential purchases, during the COVID-19 pandemic (Puiras et al., 2022). During the COVID-19 pandemic, individuals had limited opportunities to engage in leisure activities. The suspension of access to various facilities due to lockdowns may have led people to indulge in shopping as a hedonic motivation for experiencing enjoyment, arousal, or distraction (Koch et al., 2020). As for eating addiction, contrary to our study, which did not find a significant increase in addiction symptoms over the measurement points, several other studies have reported a significant increase in calorie-dense food consumption (Farello et al., 2022), BMI (Woolford et al., 2021; Yang et al., 2020), and obesity (Yang et al., 2020) among children and adolescents during the pandemic. A recent review and meta-analysis also found an increase in food addiction during periods of lockdowns (Alimoradi et al., 2022). The significant rise in symptoms of sports addiction, particularly notable at the third data collection point−1 year following the return of children and adolescents to regular school and extracurricular activities–highlights the importance of ongoing research into the enduring and complex impacts of the COVID-19 period, extending beyond its immediate aftermath. During the lockdowns, children and adolescents experienced increased sedentary behaviors (Paterson et al., 2021) due to restrictions on outdoor activities. Once these restrictions were lifted, there might have been a compensatory reaction, where the young individuals overly engaged in physical activities to make up for the previous period of inactivity, which could manifest as exercise addiction symptoms.

The research findings suggest that protective factors, such as wellbeing, social support, and secure attachment, were associated with lower levels of risk factors, including mental health symptoms and negative emotions. In turn, lower levels of these risk factors were associated with fewer behavioral addiction symptoms. The mediation model observed in this study suggested that the association between protective factors and behavioral addiction symptoms operated indirectly through mental health-related variables. This highlights the importance of mental health in understanding and addressing these behaviors. From a clinical perspective, these results suggest that interventions may benefit from focusing on mental health by reducing psychological distress and negative emotions. Strengthening protective factors, such as secure attachment and coping skills, may also contribute to prevention efforts as part of a broader approach.

In this study, future orientation, hope, life satisfaction, positive emotions, and secure attachment were protective factors against gambling, shopping, physical exercise, and eating addictive behaviors. The COVID-19 pandemic has undeniably exacerbated stressors, particularly among the youth, potentially escalating the risk of addiction. However, the presence of future orientation and hope can serve as a compass guiding them through uncertainty, enabling foresight and planning that counteract the immediacy of addictive gratifications (Du and Lyu, 2021; Wills et al., 2008).

Life satisfaction and positive emotions, often reflective of an individual's overall psychological wellbeing, may further protect against problematic addictive behaviors by fostering greater fulfillment and reducing vulnerability to external sources of gratification (Özparlak et al., 2025; Shi et al., 2025). These internal states can foster a robust psychological environment where the temptation of addiction is less appealing. Moreover, secure attachment significantly influences the development of healthy relational dynamics and the pursuit of support systems. It fosters emotional regulation, enabling young individuals to navigate stress more effectively and reducing their susceptibility to addictive behaviors (Favieri et al., 2025; Zeinali et al., 2011).

Integrating these protective factors into intervention strategies could be highly effective. For instance, programs that enhance future orientation and hope may involve goal-setting workshops, mentorship programs, and educational initiatives that emphasize long-term rewards over immediate pleasures. Similarly, fostering life satisfaction and positive emotions could be achieved through mindfulness practices, positive psychology exercises, and community-building activities that promote a sense of belonging and purpose. Such interventions, concurrently, can help alleviate psychological distress such as anxiety and depression, thereby empowering individuals to embed the protective factors optimally (e.g., Gao et al., 2021; Khazaei et al., 2017). By nurturing these elements within the youth, society can bolster their resilience and provide them with the psychological tools necessary to navigate the complex landscape of the pandemic and beyond.

Conversely, the study also found that higher levels of sensation-seeking, psychiatric symptoms, and negative emotions were associated with higher levels of behavioral addiction symptoms, including shopping, gambling, and excessive eating. The COVID-19 pandemic has likely exacerbated these risk factors, with research suggesting that addictive behaviors are often linked to self-regulation difficulties, impulsivity-related vulnerabilities, and a desire for intense experiences (e.g., Aguilar-Yamuza et al., 2024; Mancone et al., 2025). The pandemic's confinement measures, which have limited social interactions and activities, may have intensified sensation-seeking behaviors among youth, leading to an increased risk of addiction. This highlights the need for targeted interventions that address the specific challenges posed by the pandemic, focusing on enhancing self-regulation and providing healthy outlets for sensation-seeking tendencies.

Furthermore, the findings of this study show significant associations between negative emotions, psychiatric symptoms and sensation-seeking, and addictive behavior symptoms of sports, eating, shopping, and gambling. Behavioral addictions, akin to substance addictions, often function as a means to mitigate psychological distress, acting as a form of self-medication (Khantzian and Albanese, 2008). The heightened stress and anxiety induced by the pandemic may have propelled children and adolescents toward addictive behaviors as a form of solace and emotional escape. This underscores the importance of addressing the underlying emotional turmoil through therapeutic interventions and support systems, thereby providing young individuals with healthier coping mechanisms in these unprecedented times.

Our study's findings suggest a more complex relationship between social support and behavioral addiction symptoms than is often assumed in the literature. Specifically, higher perceived social support was associated with higher levels of addiction symptoms related to exercise, gambling, and eating. This finding differs from previous literature, which has typically described social support as a protective factor against addiction (e.g., Cui and Chi, 2021; Lahti et al., 2024). The unique circumstances of the COVID-19 pandemic shed light on this anomaly. With lockdowns limiting social interaction, the support of close friends became a crucial, albeit limited, outlet. Engaging in physical activities with friends, one of the few allowed diversions, or eating together during informal gatherings, may have inadvertently fostered addictive behaviors as a form of coping with the restrictions. Thus, despite the benefits of social support, it appears that during the pandemic, it may have also served as a pathway to certain addictive behaviors due to the constrained environment. In addition, in the absence of varied stress-relief options, children and adolescents might have resorted to these behaviors as coping mechanisms, and social support inadvertently facilitated rather than discouraged such activities.

The study highlighted gender-specific trends in behavioral addictions. It was observed that girl participants tended to have higher instances of shopping and eating addictions, whereas boy participants were more inclined toward gambling and exercise addictions. These findings are consistent with previous research. For example, a comprehensive review on compulsive buying behavior revealed a higher tendency for compulsive buying disorder among women (Maraz et al., 2016). During the COVID-19 lockdown in France, problematic eating behaviors such as binge eating were associated with female gender (Flaudias et al., 2020). In terms of exercise addiction, men generally exhibited greater addiction levels (Dumitru et al., 2018). Similarly, gambling addiction rates were higher among men, as evidenced by studies such as (Emond et al. 2022) and (Wong et al. 2013), with a Swedish study among adolescents showing a higher number of boys identified as problem gamblers (Claesdotter-Knutsson et al., 2022).

Although the current study did not examine mechanisms that might explain these gender differences, the impact of the COVID-19 pandemic cannot be overlooked. The lockdowns and social restrictions may have amplified existing gender-based predispositions toward certain behaviors, as individuals sought ways to cope with the stress and isolation. For example, the increased accessibility of online shopping and gambling during the pandemic might have made these activities more attractive and accessible, leading to higher levels of addiction in those already predisposed.

The observed data suggest a nuanced relationship between age and addictive behaviors. As children mature, there is a notable decrease in the propensity for shopping, exercise, and eating addictions. This could be attributed to the development of more robust self-regulation skills and a shift in interests toward more age-appropriate activities. Conversely, the rise in gambling addiction with age might reflect the increased access to gambling activities and the heightened susceptibility to risk-taking behaviors during adolescence. These findings underscore the importance of age-specific interventions in addressing various addictive behaviors in the context of the COVID-19 pandemic.

The current study has several limitations. Firstly, it relied on self-report measures from the participants. It is possible that reporting on addictive behaviors and mental health measures may have been influenced by social desirability bias. Future studies should consider incorporating parental reports and additional objective measures, such as BMI, to comprehensively assess the participants' addictive behaviors. Furthermore, the study utilized a longitudinal design with data collected at three time points. However, the pandemic's dynamic nature might have influenced changes in addictive behaviors and mental health in ways that may not be fully captured by the study's time points or duration. Additionally, the study did not measure specific variables related to the COVID-19 context, such as social isolation measures, changes in school and social environments, or whether participants or their family members were directly affected by COVID-19 infections. The omission of these COVID-19 context-specific variables may limit our understanding of the unique impact of the pandemic on addictive behaviors and mental health outcomes. Another limitation of this study is that it did not examine moderation effects, such as whether protective factors might buffer the relationship between risk factors and behavioral addiction symptoms. For instance, while higher rates of psychiatric symptoms or heightened negative emotions may increase the likelihood of addictive behaviors, substantial protective factors or a high level of resilience could potentially mitigate these risks. Future research should explore these moderation effects to understand better how protective factors interact with risk factors, offering additional insights into tailored prevention and intervention strategies. Lastly, although the present measure included psychological features commonly associated with addiction, it does not constitute a clinical diagnostic tool, and some of the reported behaviors may reflect high engagement rather than addiction *per se*. Future studies should therefore develop appropriate diagnostic tools to more accurately identify addictive behaviors among children and adolescents and better define the criteria for such behaviors (Vidal and Meshi, 2023).

In summary, the COVID-19 pandemic has profoundly affected the lives of children and adolescents, altering their routines, limiting social contact, and challenging their mental health. Faced with these unprecedented stressors and constrained psychological resources, some young individuals may have become more vulnerable to behaviors such as excessive eating, exercise, shopping, and gambling as potential maladaptive coping responses. While such behaviors may offer momentary relief, they can lead to detrimental long-term effects on mental health, functional impairments, and the potential for developing an addiction (e.g., Daglis, 2021). These findings suggest that intervention programs may benefit from emphasizing protective factors and strengthening adaptive coping strategies. A deeper understanding of these dynamics may help inform interventions aimed at reducing the risk of addictive behaviors, thereby nurturing the mental health of young people and fostering their overall resilience and wellbeing.

## Data Availability

The data is available upon request. Requests to access the datasets should be directed to ashoshani@runi.ac.il.
